# Case report: SGLT2i, transcutaneous vagus nerve stimulation, and their effects on intrarenal venous flow pattern in HFpEF

**DOI:** 10.3389/fnins.2022.999831

**Published:** 2022-09-16

**Authors:** Michiaki Nagai, Keigo Dote, Masaya Kato, Shota Sasaki, Noboru Oda, Carola Y. Förster

**Affiliations:** ^1^Department of Cardiology, Hiroshima City Asa Hospital, Hiroshima, Japan; ^2^Department of Anaesthesiology, Intensive Care, Emergency and Pain Medicine, Würzburg University, Würzburg, Germany

**Keywords:** SGLT2i, vagus nerve stimulation, intrarenal venous flow pattern, renal congestion, HFpEF, laterality

## Abstract

Renal congestion in heart failure (HF) is a predictor of the prognosis of cardiovascular disease. The effect of sodium-glucose cotransporter 2 inhibitors (SGLT2i) and vagus nerve stimulation (VNS) on renal congestion has not been reported in HF. A 77-year-old man with HF with preserved ejection fraction (HFpEF) was referred to our hospital because of poor response to loop diuretics. Echocardiography showed severe tricuspid regurgitation with dilation of the right atrium. Three months after adding SGLT2i, body weight was lost without worsening of renal function. Left and right doppler-derived intrarenal venous flow (IRVF) has been changed from a monophasic to a discontinuous pattern with a systolic interruption. One month later, he discontinued SGLT2i administration at his own discretion. In order to stabilizing autonomic balance, transcutaneous VNS (tVNS) was performed *via* left ear tragus. One hour after transcutaneous tVNS, ipsilateral IRVF has been dramatically improved from a fusional biphasic to a discontinuous pattern with a systolic interruption. SGLT2i and tVNS may be associated with renal decongestion in HFpEF.

## Introduction

A growing attention to renal congestion in heart failure (HF) is accumulating for doppler-derived intrarenal venous flow (IRVF), a reliable and feasible visual biomarker, not only as an evaluation of renal circulation, but also as a predictor of the prognosis of cardiovascular disease (Iida et al., 2016; Seo et al., 2020). Recent trials have shown that sodium-glucose cotransporter 2 inhibitors (SGLT2i) improved the prognosis of patients with HF with reduced ejection fraction (EF) (HFrEF) (Packer et al., 2020) and those with HF with preserved EF (HFpEF) (Anker et al., 2021). In patients with type 2 diabetes at high cardiovascular risk, SGLT2i was associated with slower progression of kidney disease (Wanner et al., 2016). The evaluation of IRVF is expected to guide the decongestion therapy such as SGLT2i administration.

Neuromodulation is a new treatment used in HF. An implantable device has been developed that provides autonomic regulation therapy *via* vagus nerve stimulation (VNS) (Premchand et al., 2014). This approach has an established safety profile for the treatment of refractory epilepsy or depression, and there is evidence of the potential benefits of HFrEF through multiple cardioprotective mechanisms (Hadaya and Ardell, 2020). In the CHF rat model, invasive VNS decreased efferent renal sympathetic nervous system (SNS) activity (Dibona et al., 1997). In human, SNS excitement was also non-invasively suppressed by transcutaneous low-level electrical VNS (tVNS) in the tragus (Clancy et al., 2014).

## Case report

**FIGURE 1:**
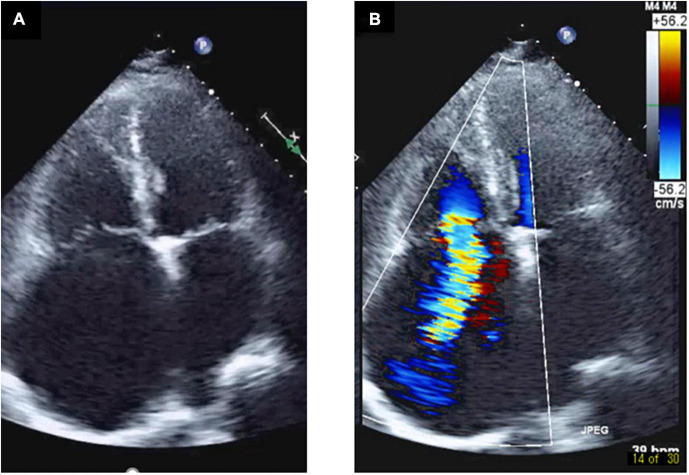
Right atrium enlargement and severe tricuspid regurgitation in a transthoracic echocardiography. Transthoracic echocardiography shows enlargement of the right atrium and a gap between the septal leaflet and anterior leaflet **(A)**, and severe tricuspid regurgitation was pointed out from the gap **(B)**.

**FIGURE 2:**
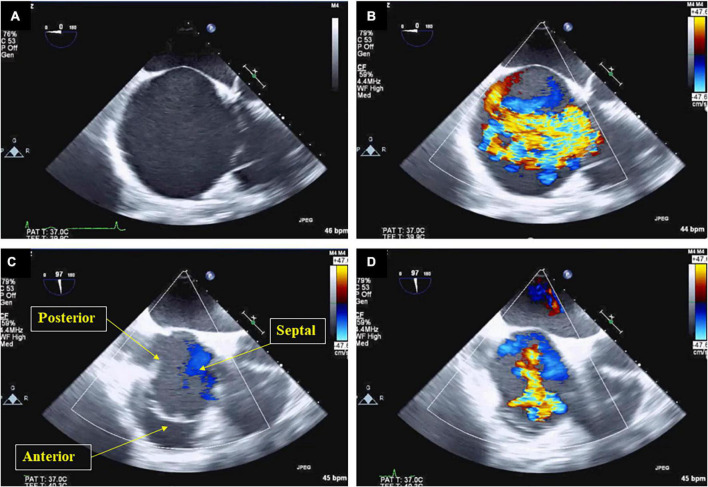
Right atrium enlargement and severe tricuspid regurgitation in a trans-esophageal echocardiography. Trans-esophageal echocardiography shows spherical enlargement of the right atrium **(A)**, severe tricuspid regurgitation swirling in the right atrium was observed **(B)**. While the anterior, septal, and posterior leaflets were indicated when the tricuspid valve was open **(C)**, a regurgitation port was found between the anterior, septal and posterior leaflets, which was also a severe finding **(D)**.

**FIGURE 3:**
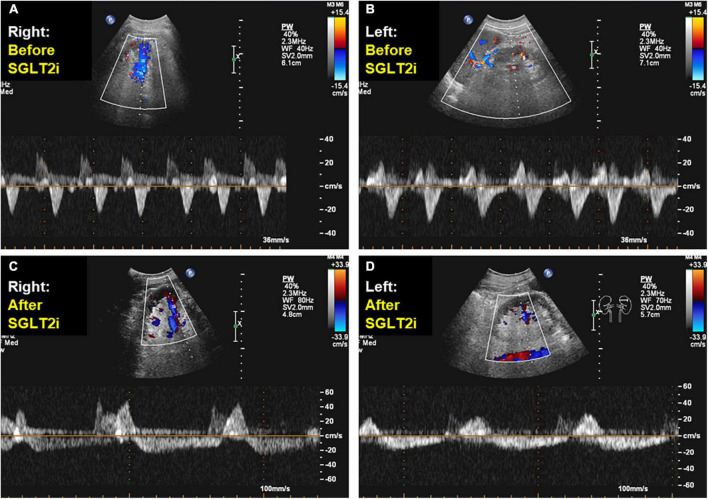
Doppler-derived intrarenal venous flows before and after sodium-glucose cotransporter 2 inhibitor administration. After adding sodium-glucose cotransporter 2 inhibitor during 1 months, in color doppler flow images from the right and left kidney, intrarenal venous flow changed from a monophasic **(A,B)** to a discontinuous pattern with a systolic interruption **(C,D)**.

**FIGURE 4:**
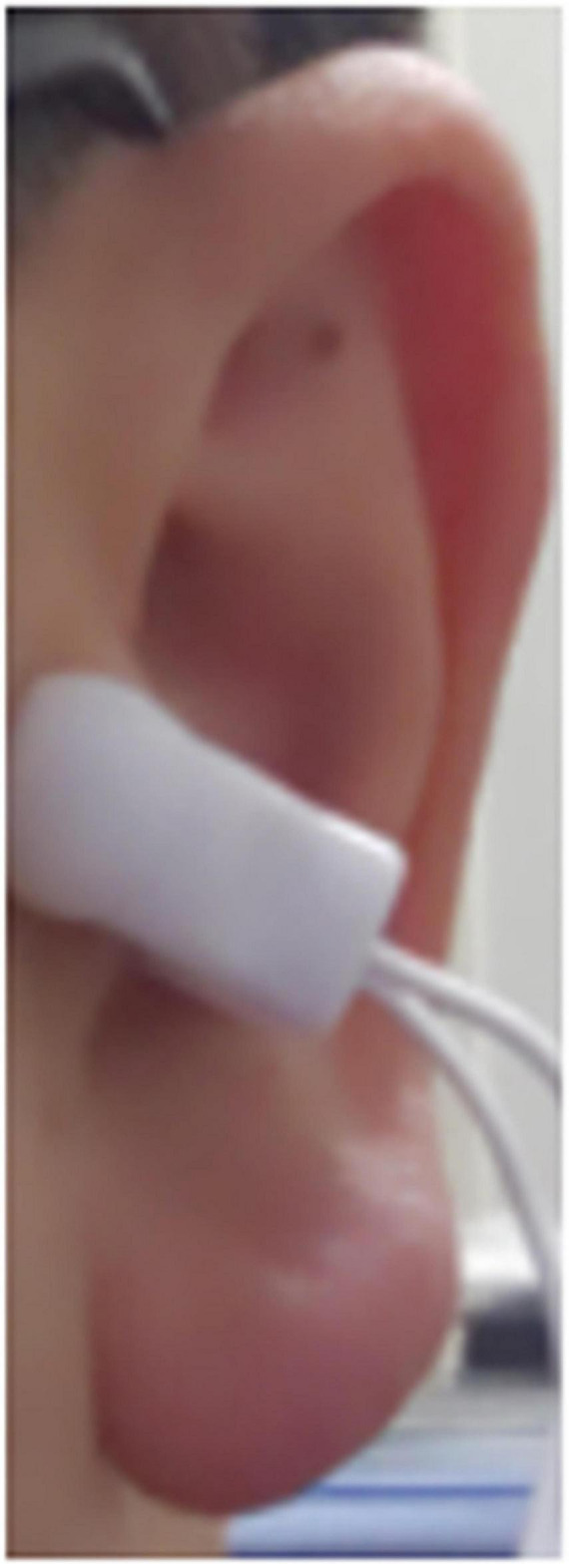
Transcutaneous electrical vagus nerve stimulation. For active stimulation, the ear clip was attached to the tragus, which is innervated by the auricular branch of the vagus nerve.

**FIGURE 5:**
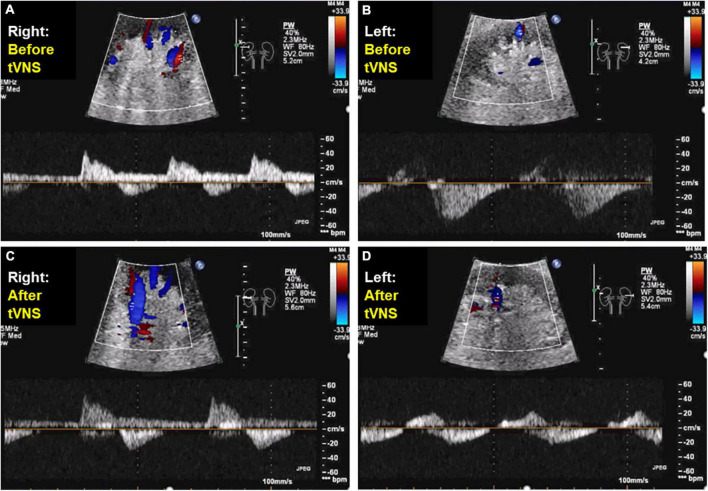
Doppler-derived intrarenal venous flows before and after transcutaneous electrical vagus nerve stimulation. After transcutaneous electrical vagus nerve stimulation during 1 h, while the right intrarenal venous flow was not changed as a monophasic pattern **(A,C)**, the left intrarenal venous flow was changed from a discontinuous biphasic pattern as one fusion wave of two waves **(B)** to a discontinuous pattern with a systolic interruption **(D)**.

**FIGURE 6:**
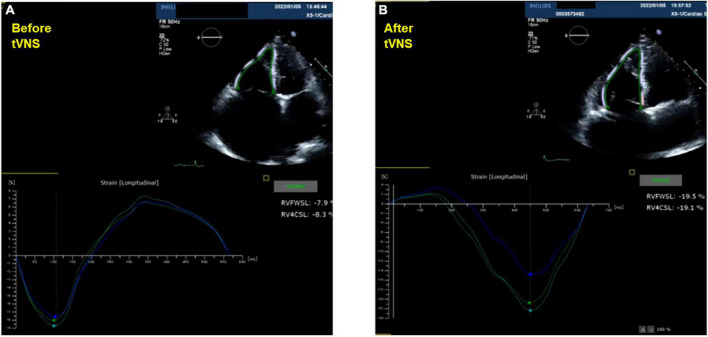
Doppler-derived intrarenal venous flows before and after transcutaneous electrical vagus nerve stimulation. Right ventricular free-wall longitudinal strain and four chamber longitudinal strain were changed before **(A)** and after transcutaneous electrical vagus nerve stimulation during 1 h **(B)**.

A 77-year-old man suffering from HFpEF was referred to our hospital on July 14. From the beginning of June 2021, edema of the lower leg, weight gain, and dyspnea during exertion increased, and the symptoms did not improve despite taking loop diuretics. While he was a social drinker, past medical history included hypertension, chronic kidney disease (CKD), obesity, type 2 diabetes and atrial fibrillation (Af). At the time of admission, consciousness was clear, blood pressure was 118/83 mmHg, and heart rate was 72 bpm. Bilateral lower leg edema as well as neck vein swelling were observed. Medication at the time of admission included oral administration of furosemide 100 mg/day, torasemide 8 mg/day, and apixaban 5 mg/day, while β-blockers and renin-angiotensin inhibitors had bradycardia and hypotension, making it difficult to continue taking them before admission. Although he had CKD, there was not hyperkalemia at the time of admission. Echocardiography showed severe tricuspid regurgitation (TR) with dilation of the right atrium (RA) (Figures 1A,B), mild mitral regurgitation, and preserved left ventricle (LV) EF in 58%. This patient met criteria for diastolic dysfunction with preserved LV systolic function (Table 1; McDonagh et al., 2021). Transesophageal echocardiography showed a spherical RA enlargement, and a regurgitation port was found between the anterior, septal and posterior leaflets, which was also a severe finding (Figures 2A–D). Because he did not accept an invasive treatment, pharmacological treatment was performed. Three months after adding SGLT2i of luseogliflozin (2.5 mg/day) without any other changes to the patient’s pharmacological therapy, body weight was successfully lost without further deterioration of renal function because the change in the estimated glomerular filtration rate was 34.8–34.5 mL/min/1.73 m^2^. Extracellular water and intracellular water were also decreased (BIA, Inbody 720, Japan Inc., Tokyo) (Table 2). In color doppler flow images from bilateral kidney, IRVF was changed from a monophasic (Figures 3A,B) to a discontinuous pattern with a systolic interruption that was close to a continuous pattern (Figures 3C,D), while TR velocity was decreased to 2.19 m/s. However, 1 month later, he discontinued SGLT2i administration at his own discretion and was diagnosed with bilateral lower leg edema. At that time, he didn’t want additional pharmacological medication. Because tVNS was expected to safely suppress SNS activity, which was considered to be a common pathophysiology for HF and CKD, with dilatation of microvasculature, stimulation of the left ear tragus (Parasym device^®^, Parasym Health, London, UK) (Figure 4) was accomplished after obtaining consent in order to stabilizing autonomic balance. One hour after tVNS (frequency of 20 Hz, pulse width of 200 ms, and the stimulation amplitude of 1 mA below the level that caused mild discomfort) (Stavrakis et al., 2020), while the right IRVF was not changed as a monophasic pattern (Figures 5A,C), the left IRVF has been dramatically changed from a discontinuous biphasic pattern as one fusion wave of two waves (Figure 5B) to a discontinuous pattern with a systolic interruption (Figure 5D). In addition, TR velocity was changed from 2.88 to 2.61 m/s, while tricuspid annular plane systolic excursion (TAPSE) was changed from 18 to 27 mm after tVNS. Moreover, right ventricular (RV) free-wall longitudinal strain (RVFWSL) and RV four chamber longitudinal strain (RV4CSL) were improved from –7.9 and –8.3% to –19.5 and –19.1% after tVNS (Figure 6). Currently, his general condition is stable without any inpatient treatment, and he is regularly visited our outpatient department.

**TABLE 1:** Objective evidence for the presence of left ventricular diastolic dysfunction.*

Parameter	
Left ventricular mass index,	81 g/m^2^,
Relative wall thickness,	0.43,
Remodeling pattern	Concentric left ventricular remodeling**
Left atrium volume index	56.7 mL/m^2^
E/e’ ratio	14.4
Brain natriuretic peptide	60.2 pg/μL***
Tricuspid regurgitation velocity	2.88 m/s

*According to 2021 ESC Guidelines for the diagnosis and treatment of acute and chronic heart failure (McDonagh et al., 2021). **The presence of concentric LV remodeling is supportive for diastolic dysfunction. ***Up to 20% of patients with invasively proven HFpEF have the values below diagnostic thresholds, particularly in the presence of obesity.

**TABLE 2:** Changes in body fluid balance before and after SGLT2i administration.

Parameter	Before	After 3 months	Change
Body weight (kg)	66.4	62.0	–4.4
Body fat mass (kg)	19.5	19.2	–0.3
TBW (L)	34.7	31.6	–3.1
ECW (L)	13.8	12.3	–1.5
ICW (L)	20.9	19.3	–1.6
ECW/TBW	0.398	0.389	–0.009
ECW/ICW	0.660	0.637	–0.023

TBW indicates total body water, ECW: extracellular water, ICW, intracellular water.

## Discussion

Attention is focused on HF renal congestion. IRVF is a reliable biomarker as a predictor of cardiovascular disease. In this case, changes in the IRVF pattern were observed in the treatment of HF with oral SGLT2i administration and tVNS. To date, few reports have examined changes in IRVF before and after HF treatment with SGLT2i or tVNS. The prognosis is suggested to be poor in the order of monophasic, biphasic, discontinuous with systolic interruption, and continuous IVRF patterns (Iida et al., 2016). The interruption was strongly influenced by an increase in each particular RA pressure (RAP) point during the cardiac cycle, rather than the average RAP level (Seo et al., 2020). Similarly, for each IRVF interruption, the existence of a specific threshold for RAP points was revealed (Seo et al., 2020).

SGLT2i causes a shift in extracellular water from the interstitial fluid (IF) space to the intravascular space to compensate for the SGLT2i-induced hypovolemia (Kuriyama, 2019). After 3 months of oral SGLT2i, the left and right IRVFs changed from monophasic to discontinuous with a single early systolic interruption in this case. A discontinuous pattern limited in the early diastolic phase, which was associated with an elevated x-descend, is associated with advanced right HF with markedly elevated RAP (Seo et al., 2020). On the other hand, a discontinuous pattern with an early systolic interruption, which is associated with an elevated a-wave, occurred in patients with RAP within the normal range (Seo et al., 2020). Thus, in this case, SGLT2i could protect kidney function by improving latent renal congestion with symptomatic HF. Assessment of IRVF might help guide decongestive therapy such as SGLT2i administration.

Neuromodulation is a novel therapy that has been used successfully in various diseases including HF (Premchand et al., 2014; Hadaya and Ardell, 2020). Excitation of SNS as well as inflammatory cytokines were suppressed non-invasively by low-level tVNS *via* the tragus (Clancy et al., 2014; Stavrakis et al., 2015). In this case, after 1 h of tVNS *via* the left tragus, ipsilateral IRVF was changed from a fusional biphasic pattern to a discontinuous pattern with a systolic interruption point. Until now, there have been no literatures that reported the relationship between tVNS and IRVF change specifically in HFpEF. A biphasic discontinuous pattern with two interruption points in both the systolic and diastolic phase, which was associated with an elevated v-wave, occurred in patients with significant TR and right ventricular dysfunction (Seo et al., 2020). Even short-term tVNS might dilate microvascular system, reduce the load on the right heart system with increased RV performance, and improve renal congestion in this patient. In this patient, TR velocity was decreased, while TAPSE, RVFWSL, and RV4CSL were improved after tVNS. Because a discontinuous pattern with a systolic interruption point was suggested to be close to continuous IRVF pattern (Seo et al., 2020), acute effect of tVNS was observed on the improvement renal congestion in this patient. Conventionally, the left tragus has mostly been selected as the preferred stimulation site due to safety concerns arising from observations during animal studies showing that right-sided VNS results in a greater degree of bradycardia (Yuan and Silberstein, 2016). While the tVNS *via* left tragus was associated with the change in the ipsilateral IRVF, the right IRVF was not changed as a monophasic pattern. Still, there is no evidence that the novel method of tVNS in the area of the left outer ear of tragus is specific for the vagus nerve system. Although our results indicate that tVNS can produce a lateralization effect on IRVF, it is not clear whether tVNS improved IRVF pattern effectively ipsilateral to the stimulated side or not (Chen et al., 2021), which reflects the lateralization effect of tVNS on renal decongestion.

## Conclusion

In this case with HFpEF, SGLT2i as well as tVNS were effectively associated with improvement of renal congestion. IRVF may provide additional tools to assess the adverse hemodynamic impact of venous congestion and may offer insights to personalize management in HF patients. Further studies will be needed to elucidate the pathophysiology underlying the relationship between SGLT2i and a shift in extracellular water from the IF space to the intravascular space, and to elucidate the pathophysiology underlying the relationship between tVNS and a hemodynamic change in right heart system in relation to renal congestion.

## Data availability statement

The raw data supporting the conclusions of this article will be made available by the authors, without undue reservation.

## Ethics statement

The studies involving human participants were reviewed and approved by the Ethics Committees of the Hiroshima City Asa Hospital (02-6-25). The patients/participants provided their written informed consent to participate in this study.

## Author contributions

MN, KD, MK, SS, NO, and CF contributed to the conception of the case report. MN, KD, MK, and NO participated in the analysis and interpretation of ultrasound. MN and CF assisted supervised the overall production of this case report. All authors have read and approved the manuscript.
